# New insight into the role of autophagy in tumorigenesis

**DOI:** 10.15698/cst2017.12.116

**Published:** 2017-12-04

**Authors:** Yongjun Tian, Linya Wang, Jing-hsiung James Ou

**Affiliations:** 1 Department of Hematology, Quest Diagnostics Inc, West Hills, Los Angeles, California, USA.; 2 Department of Molecular Microbiology and Immunology, University of Southern California Keck School of Medicine, Los Angeles, California, USA.

**Keywords:** autophagy, mitophagy, hepatic cancer stem cells, hepatocarcinogenesis, p53, PINK1

## Abstract

Autophagy plays an important role in maintaining cellular homeostasis. Its dysfunction can cause many diseases, including neurodegenerative diseases, metabolic diseases and cancer. The role of autophagy in carcinogenesis is complex, as it was shown to have pro-tumorigenic functions in some reports, but anti-tumorigenic functions in others. By using mice with hepatocyte-specific knockout of *Atg5*, a gene essential for autophagy, we had previously demonstrated that impairing autophagy in hepatocytes would induce oxidative stress and DNA damage, followed by the initiation of hepatocarcinogenesis. Interestingly, these mice developed only benign tumors with no hepatocellular carcinoma (HCC), even after they were treated with the carcinogen diethylnitrosamine (DEN), which induced HCC in wild-type mice. Our recent studies indicated that the inability of mice to develop HCC when autophagy was impaired was at least partially due to the activation of the tumor suppressor TP53, which suppressed the expression of NANOG, a transcription factor critical for the self-renewal and the maintenance of cancer stem cells (CSCs).

TP53, commonly known as p53, is an important tumor suppressor. Its increased expression was observed not only in the liver tumors of our *Atg5*-knockout mice but also in the lung tumors of *Kras* and *Atg7* double-knockout mice and in the pancreatic tumors of mice with *Atg7* knockout in the pancreatic tissue. The same as *Atg5*, *Atg7* is an autophagy-related gene that is essential for the initiation of autophagy. In these mice with *Atg7* knockout, the lung tumors or pancreatic tumors detected were also benign tumors. The analysis of liver tumors isolated from *Atg5*-knockout mice and control mice treated with DEN revealed a significant loss of CD133 and CD49f double-positive cells in the tumors of *Atg5*-knockout mice. CD133 and CD49f are markers of CSCs. These observations indicated that autophagy might promote tumorigenesis via the suppression of p53 expression and the induction of CSCs.

The effect of autophagy on CSCs was further confirmed in our recent studies using HepG2 cells, a human hepatoblastoma cell line. We found that, when autophagy was induced, the population of CD133^+^ HepG2 cells increased, and when autophagy was inhibited, the population of CD133^+^ HepG2 cells decreased. Unlike CD133^-^ HepG2 cells, CD133^+^ HepG2 cells could self-renew and proliferate in low-attachment plates, which is an important characteristic of CSCs. These CD133^+^ HepG2 cells could also form tumors when they were grafted into immunodeficient nude mice. The effect of autophagy on CD133^+^ cells was dependent on p53, as it had no effect on Huh7 cells, which express a defective p53, and Hep3B cells, which do not express p53. Both Huh7 and Hep3B are human hepatoma cell lines. The effect of autophagy on CD133^+^ cells of these hepatoma cells could be restored when functional p53 was expressed in these cell lines. We had further examined how p53 might regulate CSCs and found that p53 phosphorylated at serine-392 (S392) could bind to the *NANOG* promoter. This binding prevented the transcription factors OCT4 and SOX2 from binding to and activating the *NANOG* promoter, resulting in the suppression of NANOG expression. Since we also found that the inhibition and the induction of autophagy could increase and decrease the phosphorylation of p53 at S392, respectively, these findings together explained how autophagy regulated CSCs in a p53-dependent manner.

Mitophagy is a selective autophagy that specifically removes mitochondria. It plays an important role in the quality control and homeostasis of mitochondria. PINK1 is a PTEN-induced kinase and important for mitophagy. Interestingly, the same as autophagy, the inhibition of mitophagy could increase the levels of p53 and its S392 phosphorylated form and reduced CSCs, and the induction of mitophagy had the opposite effect on p53 and CSCs. As mitophagy by itself was sufficient to affect p53 and CSCs, these results indicated the effects of autophagy on p53 and CSCs were likely mediated by mitophagy. To further investigate the relationship between mitophagy and p53, we examined the subcellular localization of the S392 phosphorylated form of p53. Although this phosphorylated form of p53 was found to localize predominantly in the nucleus when mitophagy was inhibited, it was found to co-localize with mitochondria or in their vicinity when mitophagy was induced, suggesting the possible removal of p53 with its associated mitochondria by mitophagy.

Due to its importance in mitophagy, we also investigated the relationship between p53 and PINK1. To our surprise, we found that the knockdown or the over-expression of PINK1 had no effect on the overall p53 protein level, but they suppressed and enhanced the phosphorylation of p53 at S392, respectively. This result indicated that PINK1 might be the kinase that was responsible for the phosphorylation of p53 at its S392. This possibility was confirmed by the two following observations: first, PINK1 purified from HepG2, Huh7 and Hep3B cells could phosphorylate recombinant p53 at S392 *in vitro*; and second, the incubation of recombinant PINK1 and p53 led to the phosphorylation of p53 at S392.

Recent studies, including ours, provided important information for understanding the role of autophagy in carcinogenesis. Autophagy can act both as a tumor suppressor and a tumor promoter. In our studies, we demonstrate that autophagy, or more specifically mitophagy, is important for removing damaged mitochondria. If autophagy/mitophagy is impaired, dysfunctional mitochondria will accumulate, which will lead to the increase of oxidative stress and DNA damage and the initiation of hepatocarcinogenesis. However, after the initiation of hepatocarcinogenesis, autophagy/mitophagy is also required for removing p53 to enhance the expression of NANOG and the expansion of CSCs for the malignant transformation of benign hepatic tumors into HCC. p53 is recruited by PINK1 to mitochondria and apparently degraded together with its associated mitochondria by mitophagy. If autophagy/mitophagy is impaired, p53 phosphorylated by PINK1 cannot be retained on mitochondria and is translocated into the nucleus where it suppresses the expression of NANOG to reduce the stemness and the self-renewal ability of CSCs, and hence the suppression of hepatocarcinogenesis. The roles of autophagy/mitophagy in hepatocarcinogenesis are illustrated in **Figure 1**. Although our recent studies were focused on HCC, it is possible that autophagy/mitophagy may also have similar effects on the CSCs of other cancers.

**Figure 1:**
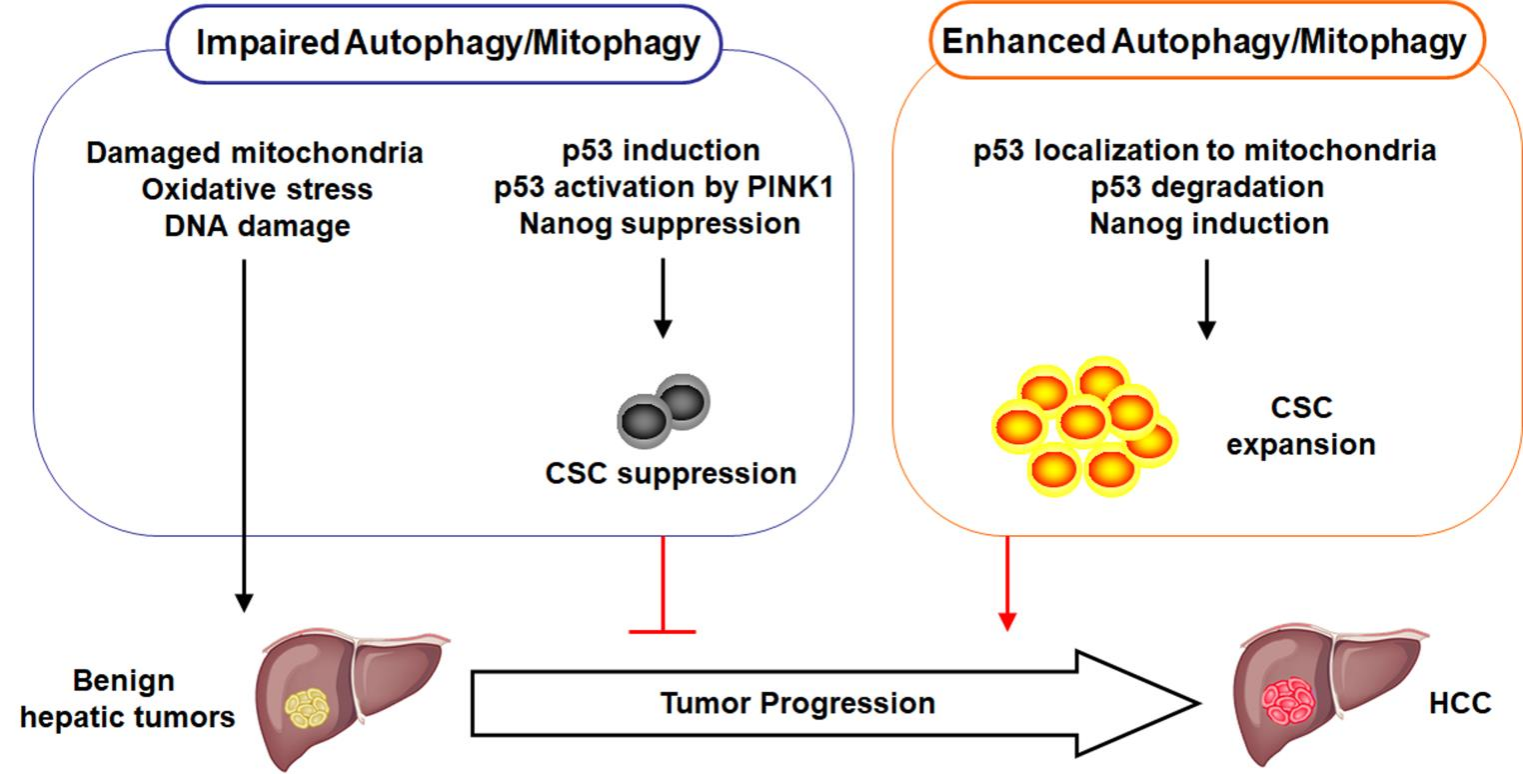
FIGURE 1: Illustration of the roles of autophagy and mitophagy in hepatocarcinogenesis. When autophagy or mitophagy is impaired, damaged mitochondria accumulate in the cell, resulting in increased oxidative stress and DNA damage and the initiation of hepatocarcinogenesis. At the same time, this impairment in autophagy or mitophagy induces the expression of p53, which is further activated by PINK1 to suppress the expression of NANOG and the production of CSCs. Consequently, the progression of benign hepatic tumors into HCC is inhibited. In contrast, when autophagy/mitophagy is enhanced, p53 is associated with mitochondria and subsequently degraded. This leads to the induction of NANOG and the expansion of CSCs to promote the development of HCC.

